# Cognitive Structures of Space-Time

**DOI:** 10.3389/fpsyg.2020.527114

**Published:** 2020-10-21

**Authors:** Camilo Miguel Signorelli, Selma Dündar-Coecke, Vincent Wang, Bob Coecke

**Affiliations:** ^1^Department of Computer Science, University of Oxford, Oxford, United Kingdom; ^2^Cognitive Neuroimaging Unit, INSERM U992, NeuroSpin, Gif-sur-Yvette, France; ^3^Center for Educational Neuroscience/Department of Psychology and Human Development, University College London, London, United Kingdom

**Keywords:** causal cognition, causal structure, causality, space-time, compositionality

## Abstract

In physics, the analysis of the space representing states of physical systems often takes the form of a layer-cake of increasingly rich structure. In this paper, we propose an analogous hierarchy in the cognition of spacetime. Firstly, we explore the interplay between the objective physical properties of space-time and the subjective compositional modes of relational representations within the reasoner. Secondly, we discuss the compositional structure within and between layers. The existing evidence in the available literature is reviewed to end with some testable consequences of our proposal at the brain and behavioral level.

## 1. Introduction

This article posits a hierarchy in the cognition of spacetime, analogous to a “layer cake” structure, where layers correspond to different aspects of causality. The foundations of the layer-cake structure are derived from physical accounts of causality, supported by a brief mathematical background. The proposed hierarchy acknowledges that neither space nor time can be accessed directly; we can only glean their structures by observing and interacting with objects among events. Therefore, the natural question is how we establish coherent models of spacetime.

Toward an answer, the present paper proposes that cognitive models are hierarchical, where lower layers encode structurally simpler data than higher ones, and the structure of spacetime emerges from mutual constraints between layers.

We take the most primitive layers to be topological, which refers to whether objects and events are “connected.” Topology does not distinguish between the types of the lines (e.g., curved or straight); only connectedness—however defined—and its absence, disconnectedness, need be perceived. In the perception of spatial-temporal entities, connectivity and disconnectivity compositionally characterize more complex features such as being “before,” “after,” “in front,” “behind,” “having holes,” “discreteness,” etc.

A more complex, computationally dense and higher up layer might construct metric spaces and Euclidean structure. An example of a constraint between topology and metrics that may arise in some setting is “*objects are connected if and only if they have zero distance from each other.”*

Investigating the cognitive structures of space-time governing causal cognition is central to the understanding of a general theory of intelligence in humans and in artificial beings. Nevertheless, in psychology, research lags in providing a concise and systematic review for the correspondences between empirical causal structures and spatial-temporal cognition.

Beyond that, the layer-cake organization of spatial-temporal structures are preserved among other fields, such as physics, mathematics and also computer science, leading to a natural hierarchical organization from topological space (less complex), to metric spaces (more complex). In the following sections, we explore this toy model in the context of physical causal structures (section 2), then provide psychological models (section 3) and continue with a discussion of its implications in a wider context (section 4).

## 2. Layers of Structure in Physics

In Physics, the analysis of the spaces representing potential states of physical systems often takes the form of a layer-cake of increasingly rich structure. The layer-cake is not merely a mathematical decomposition, but is informed by some conceptual underpinning: such as how agents interact with the subject matter, and more specifically, how the subject matter enables/restricts this interaction, or how the subject matter interacts with itself.

**Figure 1:**
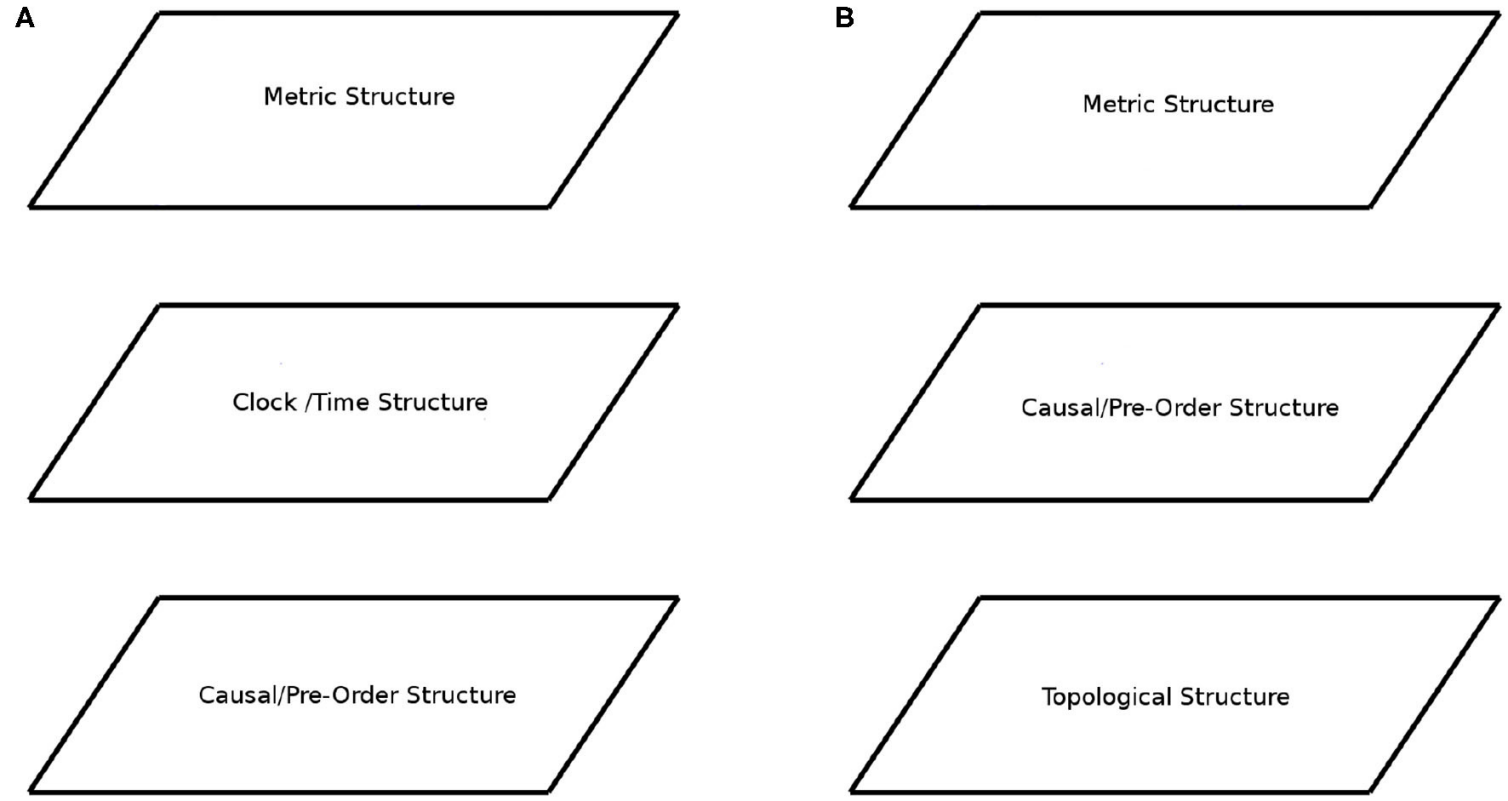
Layer-cake structure. **(A)** The layer structure of Relativistic Space-Time. **(B)** The layer structure of Causal process theories and the hypothesized layer Causal structure for Cognition.

A first example is the analysis of relativistic space-time structure as for example in Geroch (2013) and Ehlers et al. (2012). Here, the levels arise from how agents interact with space-time. In Geroch (2013), like in many other such approaches, the first layer is called *causal structure* (Figure 1A). It arises from the light-cones that specify which points of space-time (in the future) the agent can affect, and which points of space-time (in the past) the agent can be affected by. Mathematically, these light-cones give rise to a partial order (*P*, ≤), where for *a, b* ∈ *P* we have *a* ≤ *b* if space-time point *a* can affect space-time point *b*. Often this partial order is taken as a starting point for the development of new physics, for example, when studying quantum causality (Fritz, 2014; Henson et al., 2014), and even when crafting theories of quantum gravity (Bombelli et al., 1987; Sorkin, 2003). A second layer arises from the notion of a clock (Figure 1A), which measures the progress of time and hence provides a temporal metric structure atop the partial order of events. Next comes the full space-time metric, followed by dynamical data, among others.

Moving from relativity to quantum theory (QT), following John von Neumann (von Neumann, 1932; Birkhoff and von Neumann, 1936), the first layer is again a partial order, where ordering encodes entailment with respect to agents observing properties of quantum systems, that is, *a* ≤ *b* if observation of property *a* guarantees observation of property *b*. The following layers include conceptually informed universal algebraic equational structure (Piron, 1976). Note that also the entailment relationships can be viewed as a form of informational/epistemic causal structure, as it involves a guaranteed observation given a premise. This branch of quantum theory has mostly vanished from current activity within physics, but has been adopted within psychology in the field of quantum cognition (Busemeyer and Bruza, 2012).

Much more recently, in the category-theoretical analysis of quantum theory (Abramsky and Coecke, 2004; Coecke and Duncan, 2011; Coecke and Kissinger, 2017), rather than the interaction of agents with the subject matter, the lower levels of the layer-cake are informed by how the subject matter interacts with itself. This lowest level is fundamentally *topological*, and more specifically, what topologists call low-dimensional topology (in fact, as low-dimensional as its gets). The structure only expresses what is connected and what is not, without bringing any other geometric notions into play. In this approach, explicit graphical wiring at once formulates and represents connectivity, so it suffices to understand the concept of “wire” to understand this lowest layer of quantum theory (Figure 1B). This, in fact, leads to an alternative justification for having this particular layer as the basis: wires are, *a priori*, conceptually primitive for human reasoners (Coecke, 2005, for the indication from the title, namely “Kindergarten quantum mechanics”). An educational experiment is expected to take place during 2020 (see Coecke, 2009), aiming to show that quantum theory presented in topological terms would enable high-school students not only pass a graduate-level quantum theory exam, but even outperform university students who are taught the conventional presentation.

Within the topological approach, the notion of causality has been proven to be equivalent to the relativistic notion of causality (Kissinger et al., 2017). Thus causality can be formulated higher up in the layer-cake (Coecke and Kissinger, 2017), synthesized and restrained by more primitive data (Figure 1B). In fact, there are multiple presentations on the move from lower topological level to full-blown quantum-theory, cf. Coecke and Kissinger, 2017; Selby et al., 2018, but the topological level is always the beating heart of this approach. As it turns out, natural language is governed by exactly the same topological structures, the reason being that the structure of grammar itself (Lambek, 2008), exactly matches the topological structures of QT (Coecke, 2013, 2017). Furthermore, even more general cognitive models appealing to a wide range of human senses have been shown to be governed by the same structures (Bolt et al., 2018). The starting point here were Gärdenfors (2014)'s conceptual spaces, which aim to closely resemble human senses, and the interaction of these senses is again governed by basic topological structures.

## 3. Layers of Structure in Cognition

According to previous considerations, cognition may mirror the physical structures of spacetime, or the physical structures suggested by human theories may only reflect a basic cognitive structure of human thinking^1^. Independently of these two options, the layer-cake structures given by physical theories seem to be present in our developmental understanding of spatial and spatial-temporal structure (section 3.2). Therefore in this section, a layer-cake model is discussed as hierarchical levels of cognitive complexity, inheriting, to some extent, all the mathematical properties coming from previous developments in physics DisCoCat/InConcSpec (Coecke et al., 2010; Bolt et al., 2018), without having to develop a new one.

The layer-cake hypothesis addresses a gap in the ongoing neurocognitive debate concerning the—as Bellmund et al. (2018) argue, central—role of spatial-temporal cognition, topology, and metrics in high-level cognition. Direct correlates of euclidean space and time have been identified in neural representation (Moser et al., 2015; Tsao et al., 2018). However, as Buzsáki and Llinás (2017) and Buzsáki and Tingley (2018) observe, the reasoner only receives information concerning distance and duration, reflected in a succession of neuronal events that may not correlate with any space-time representation. This spurs a search for model-building and inferential explanations of how direct neural correlates to space and time arise from sense data, which the layer-cake hypothesis may potentially provide a framework for. Bottini and Doeller (2020) suggest that any such framework goes toward explaining a general propensity of the mind to create low-dimensional internal models. Promisingly, Haun and Tononi (2019) have derived mathematical models demonstrating that brain areas with grid-like connectivity are sufficient to entertain the topological and causal structures necessary for subjective spatial experience. So the layer-cake hypothesis, in concord with all parties of the debate, could serve as a missing link between the mechanical, theoretical, and phenomenal aspects of spatial-temporal cognition.

### 3.1. Topological Layers of Cognition

The model presented here is a general framework to develop specific implementations according to requirement. The main ingredients are the division/synthesis of causal structure in terms of more primitive structure, and organizing these composite structures into layers corresponding to constraints and affordances of causal relations, and the developmental order.

**Figure 2:**
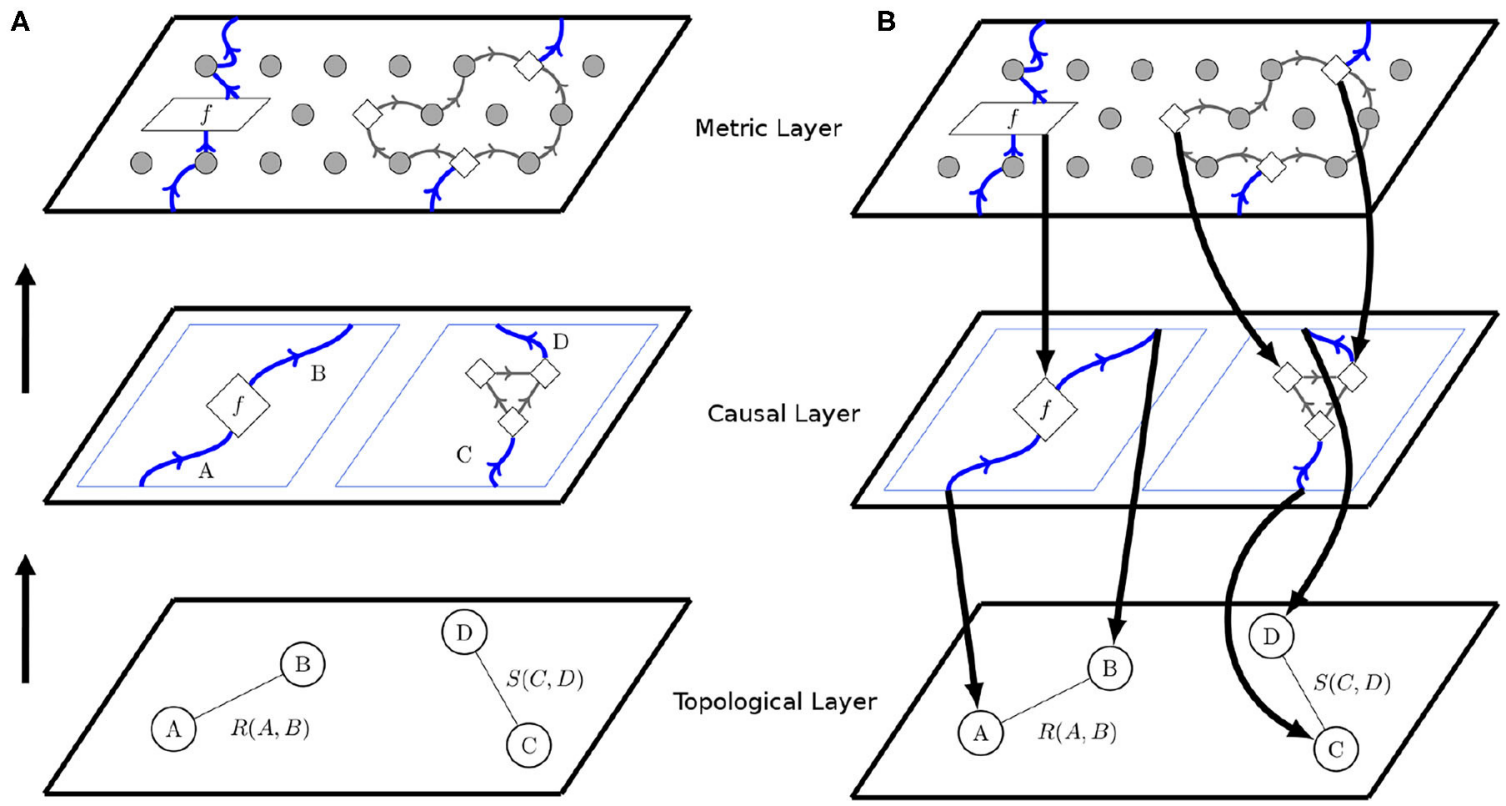
Layer structure for cognition. **(A)** Topological Structure is relational and increase in complexity as we rise through the layers. In the topological layer, circles represent objects and wires topological relations between objects. Then, causal structure, in the form of process theories is build on top of topological relations from the lower layer. In this second pre-order or basic causal layer, objects become represented by wires and causal relations by processes. Finally, the metric layer adds metric structure, which elaborates causal structure in a suitable, spatial and temporal setting. This condition is given by the black dots in the figure. **(B)** This layer division generates a hierarchical structure, where higher layers are structurally constrained by the data of lower layers as well as they can influence part of the lower configuration.

We propose that the first layer compounds Topological relations, and consequently, that comprehension of causal relations across space and time prioritizes topological structures. It implies that early or primitive forms of causal cognition and specifically spatial cognition would not be highly conceptual, only involving simple notions of proximity, separation, order, enclosure, connectivity, and boundedness. As discussed later, such conceptualization may be through non-symbolic category formation where subjects have restricted access to verbal codes: for example, fundamental ideas about space are developed in infancy by motor and perceptual mechanisms and rely strongly upon sensory/perceptual data. Diagrammatically, two objects *A* and *B*, are topologically related if there is an event that connects them, which is defined by the relation *R(A,B)*. These connections are usually described by wires and objects by nodes. Under this notation, wires are relational events and circles are static objects (Figure 2).

The relation events *R(A,B)* and *S(C,D)* connecting the objects of cognition described by *A, B, C*, and *D* correspond to fundamental and basic notions, that eventually lead to the understanding of spatial relations. Later, other types of relation emerge, such as the effects between objects, which correspond to object interactions across primitive notions of time. These interactions define processes notated by boxes, such as *f* . More specifically, such interactions may correspond to a causal processes according to a partial order relation (Figure 2A). In other words, the object *A* and *B* become causally related systems under the partial order, written *A* ≤ *B*, meaning in the abstract that information flows unidirectionally from *A* to *B*, thus defining a second layer of structure upon systems. Notably, causal relations defined in this way among objects are not necessarily unique, as exemplified by the case of *C* and *D*. Following the notation from previous works (Coecke and Kissinger, 2017), now wires become objects/systems and boxes the causal processes among them (Figure 2A).

Empirically, we abduct events from *observations* of relational spatial properties. In contrast, *processes* may encompass unobservable intervening dynamical factors (e.g., forces), which need to be constructed or reconstructed in further levels of complexity: processes correspond to abstract components of mechanisms. Therefore, the second layer would correspond to the representational/relations space associated with causal interactions, governed by the partial order relations mentioned above. We hypothesize that the gradual emergence of concepts, syntax, grammar would be associated with such higher layers, as these permit representation and reasoning with counterfactual and imaginary phenomena not immediately constrained by past experience and direct perception.

A consequence of this division is that constraint-satisfying structure on any layer, in turn, places constraints on how further layers are defined. Viewing foundational layers as abstract schema or cognitive resources (and their neural realizations) shapes the modes of access to that structure, constraining how relations take place in that schema. For instance, when we take processes in spacetime to be mutually exclusive, we can begin to fill in complex narratives. If we know that a battle and a wedding took place in the same valley, mutual exclusivity of processes and linear temporal ordering allow us to raise a fruitfully constrained set of alternative models: either the battle came before the wedding, or *vice versa*.

Hence, any layer may be viewed as an abstract space upon a lower layer, the higher further specifying instances of structure compatible with those of the lower (Figure 2B). In Figure 2A, the higher layer carries the particular refinement of metric structure. The precise nature of cognitive metric structures is a question for future research, and not our chief concern here. No matter the metric, according to the layer-cake model, representation and reasoning in metric spaces is more computationally intensive than in topological spaces, because higher layers carry a greater informational capacity than lower ones, and carry more constraints and affordances for the reasoner to navigate.

These emergent hierarchies are subjective to the reasoner, and not an objective feature of reality: hence, we can speak distinctly of perceived vs. objective causality. In other words, while the seemly real characteristics of spacetime affect how we conceptualize spacetime, our conceptualization in turn dynamically constrains and directs further conceptualization.

Finally, a word of caution when interpreting the topological hypothesis as stated above is that different conceptions of causality and topology exist, as these are not uniquely defined concepts across disciplines, and not even in pure mathematics, where a field like topology has several very different branches of study that are qualitatively different. For example, taking path-connectedness as the primitive—where one identifies possible paths that one can take between points in space—will cause one to identify all points on the surface of a table as “*essentially the same*,” whereas homology theory—where one identifies the characterizing holes of a structure—will cause one to treat drinking mugs and donuts as “*essentially the same*.” The layer-cake model accommodates any and all particular formulations of topology, as it is synthetic: the fundamental ingredient of defining higher structures atop lower ones remains in play.

### 3.2. Supporting Evidence for the Layer-Cake Structure

Developmental studies are in accord with the layer-cake hypothesis. Evidence supports the notion that topological properties, representing the earliest/primitive forms of distance-duration relations, are available initially through a nonverbal category formation, even where young children have restricted access to verbal codes (Dündar-Coecke et al., 2020). Using linguistic and non-linguistic tasks (Piaget, 1959) (see also Piaget and Inhelder, 1971) pioneered the argument that infants' perceptual space is qualitatively different from that of adults. At the beginning, fundamental spatial concepts are not Euclidean, but topological, which involves some concepts such as proximity, separation, order, enclosure, long before it becomes metric. This suggests that the infant's space must be quite fluid, not objective, nor occupied by rigid shapes or sizes.

Studies of adult cognition also acknowledge this fluency in cognitive structures. In Biederman and Cooper (1991) and Biederman and Cooper (1992) study, although participants were presented with contour-deleted pictures, they completed perceptual stimuli in the absence of size, location, or orientation information, highlighting humans' ability to recognize objects independent of Euclidian spatial features in a more abstract fashion. While these results suggest a potential primacy of topology over more complex data, research establishing cognitive mechanisms involved in conceptualization of topological and metric properties also provides consistent evidence that people cannot act within, or orient themselves to their environments unless provided spatial and temporal information constituting their physical reality (Han et al., 1999; Müsseler, 1999; Chen, 2005).

Topology's fundamental role in understanding space is supported by theoretical grounds in neuroscience: Marr (1982) posits a sophisticated *motion correspondence process* in the perception of an entity through time, simple topological transformations also enable observation of apparent motion (Chen, 1982, 2005; Ogmen and Herzog, 2010). Rock and Palmer (1990) stress the law of “connectedness” in early perceptual analysis, and the topological perception hypothesis suggests that shape-changing transformations experienced in the phenomenal world rely on topological transformations, for example, projected in retina with the aid of three kinds of topological properties: connectivity, the number of holes, and the inside/outside relationship.

Another strand of work emphasizes the role of selective attention as a strategy to bias continually registerable spatial-temporal attributes, and hence increase control in processing capacities through top-down neural connections (Kastner and Ungerleider, 2000). In fact, neuroimaging studies have shown that a number of mechanisms can contribute to attentional orientation to moving targets (Doherty et al., 2005; Shimi et al., 2014), with a prevailing view that perceptual organization (topological) likely to occur before feature analysis (metrics). Chen (1982) reports a series of experimental findings showing the precedence of topological feature detection in the visual system, further supporting the view that topological features form conceptual foundations. Pomerantz's configural superiority effect supports this hypothesis (Pomerantz, 1981; see also Todd, 1998), by adding that features can be observed even in response to stimuli that are not fully configural, as configural information is already present at early stages of visual hierarchy (see also Fox et al., 2017, for neural evidence).

Limited knowledge in furthering these discussions urges us to swing the pendulum back to the infant studies, which are highly informative regarding the detection of primitive forms of spatial-temporal properties. Infants appear to show sensitivity to moving objects along “continuous” paths, and also pay attention to interactions only if they are causally in contact (see Leslie, 1984; Leslie and Keeble, 1987; Spelke et al., 1992, 1995a; Spelke, 1994, see also Darcheville et al., 1993, for how infants learn about space as a function of the temporal intervals). However, they seem to find it difficult to relate objects based on non-causal qualities, such as colors, forms, edges, or surfaces (Kellman and Spelke, 1983). Instead, they show a tendency to rely on simple forms of spatial-temporal information to distinguish different types of objects and events (see Slater et al., 1994; Spelke et al., 1995b; Needham et al., 1997; Wilcox and Baillargeon, 1998; see also Kaufman et al., 2003, for evidence how spatial-temporal stimuli are processed by different visual streams). These studies propose consistent evidence for the early sensitivity to topological spatial-temporal features such as continuity and connectivity in causal contexts. Although maturation in use of these representations are accompanied by conceptual development, humans are multimodal reasoners; most implicit spatial-temporal qualities are more akin to sensations and do not necessarily have to be available to communication (Tolmie and Dündar-Coecke, 2020). This may explain the consistency between adult and infant data.

The early fundamental ideas about space-time develop largely by embodied motor and sensory activities. Young children experience the most primitive spatial-temporal properties via observing, touching, and moving their/others' bodies. The development of symbolic cognitive resources, such as memory and language, enables spatial-temporal properties to become more representational, allowing children to mentally evoke objects in their physical absence. Understanding of or paying attention to metrics and Euclidean structures emerge as a function of the development of these internal and external resources and models. For instance, a child learns how to stack the smaller object into the big ones, or improve projective and perspective taking skills gradually. *Contextual* consistency of spatial models appears to develop later than spatial models of individual closed objects. For example, at early stages, children are likely draw a human being bigger than e.g., a house in size, while the orientation of both human and house may/not respect gravity, and relative placement of appendages and windows all correct for both human and house. The primary context in which size consistency is obtainable is the embodied motor-sensory paradigm: at the same physical distance from a human and a house, the human image may have a smaller angle of subtension in the infant's field of vision.

Therefore, developmental literature underlines the myriad ways in which spatial-temporal properties are experienced and employed in service of causal cognition, in accord with the layer-cake hypothesis where causal relations are predicated upon spatial-temporal foundation layers. The most studied spatial-temporal attributes in causal cognition literature are properties high in the layer-cake: distance, duration, velocity, and spatial-temporal incongruences (Bullock and Gelman, 1979; Siegler and Richards, 1979; Wilkening, 1981; Bullock et al., 1982; Wilkening and Cacchione, 2011). These studies sample either children or adults, and a comparison between these and early infancy studies implies that the more children/humans are able to utilize spatial-temporal properties in Euclidian fashion, the better they can acknowledge causal relations. Although the grasp of causal relations requires the organization of connections across space and time in topological sense and this is critical for visual function at any age, the genuine understanding of cause-effect relations matures when we define the richer causal geography of spacetime.

## 4. Conclusions and Future Research

The layer-cake hypothesis provides a meta-model of spacetime cognition. The main argument of this conceptual model is that spatial and temporal qualities increase in their complexity across mutually constraining layers of description, ranging from the topological to metric, temporal, and causal, for models of physical or virtual/abstract spaces. It is the layer-cake taken as a whole that can be considered the full model. The hierarchical organization of layers is a novel form to study this complexity of the spatial-temporal relations in both physics and psychology, providing rich enough model to capture not only the interaction of multiple dimensions of abstractions, but the internal dynamics of constructing cognitive models from empirical data, fed by the reciprocal interactions between perception, action, and reasoning about space, time and causality.

The layer-cake hypothesis is adaptable but crucially for science, defeasible, as it must always be instantiated to provide concrete models. These instantiations compatibly formalize a broad range of current approaches to cognition of causality across space and time. Previously, Newcombe and Shipley (2015) and Uttal et al. (2013) studies underlined how the intrinsic/extrinsic and static/dynamic relations between entities inform us about the characteristics of spatial elements, which may be modeled as graphical calculi on suitably encoded layers of a layer-cake. Developmental origins of thinking about past, current, future situations (Friedman, 2003; McCormack and Hoerl, 2005), either in segmented, speeded, or imagined protocols (Dündar-Coecke et al., 2020) may be formalized in the physicist's language of logics upon partial orders on events, again amenable to graphical and layer-cake methods of representation and reasoning. Layer-cake models are well-suited to novel developmental studies in calibration and approximation of spatial-temporal attributes on virtual displays (Dündar-Coecke, 2019), where the spatial environment is distanced from the young reasoner by a layer of abstract representation, as layer-cakes have tunable levels of abstraction built-in.

On the theoretical side, our perspective aims to generate a new interdisciplinary semantics for spatio-temporal cognitio interwoven with theoretical physics. In conjunction with experimental phases, if the layer-cake structure deduced from theoretical physics is shared or preserved in the structure of spatio-temporal cognition, we can shed light on those structures using recent mathematical tools that deal with physical space-time and causality. Throughout, we expect to use axiomatic process-theoretical tools which are currently applied for causal relationships in physics (Coecke and Kissinger, 2017; Kissinger and Uijlen, 2017). This approach will allow us to describe the nature of spatio-temporal experience in the form of interacting processes, following similar strategies already implemented for language and cognition (Coecke et al., 2010, 2018; Coecke, 2013; Bolt et al., 2018).

On the experimental side, one can ask about the neural and behavioral implications of our axiomatic models. For example, if we establish the presence of distinct but cohesive competencies for different aspects of spatial cognition and experience, a subsequent question is to ask where does the layer-cake find expression? The question of whether this paradigm finds implementational reality inside brains (as suggested by Signorelli, 2018; Signorelli and Meling, 2020) and the discussion of the feasibility of layer-cake models in terms of neural structure will form part of further extensions to this program. More broadly, we may unlock spaces of questions for developmental and evolutionary biology, to further our understanding of how agents arise in space-time and vice versa.

## Author Contributions

CS conceptualization and visualization the model, writing the original manuscript, and editing subsequent versions. SD-C conceptualization the model and wrote and edited the manuscript. VW conceptualization the model and edited subsequent versions. BC conceptualization the model and writing manuscript. All authors contribute to the original hypothesis and discussions.

## Conflict of Interest

The authors declare that the research was conducted in the absence of any commercial or financial relationships that could be construed as a potential conflict of interest.

## Footnotes

^1^That issue together with the possible neural realization will be discussed elsewhere.
